# Exploring the psychometric properties of the Persian Depression Anxiety Stress Scale for Youth (DASS-Y): factor structure and reliability in Iranian children and adolescents

**DOI:** 10.3389/fpsyg.2024.1452878

**Published:** 2025-01-15

**Authors:** Mohammad Javad Shabani, Banafsheh Gharraee, Komeil Zahedi Tajrishi

**Affiliations:** Department of Clinical Psychology, School of Behavioral Sciences and Mental Health (Tehran Institute of Psychiatry), Iran University of Medical Sciences, Tehran, Iran

**Keywords:** DASS-Y, depression, anxiety, stress, psychometric properties, children, adolescents, Persian

## Abstract

**Background:**

The Depression Anxiety Stress Scale for Youth (DASS-Y) is a self-report instrument recently developed to evaluate negative emotional states in children and adolescents. However, the Persian version’s factor structure and psychometric properties have yet to be investigated in Iranian youth. The study aimed to assess the factor structure and reliability of the Persian DASS-Y in a sample of Iranian children and adolescents.

**Methods:**

The study recruited 1,277 children and adolescents, 703 (55.1%) being girls and 574 (44.9%) being boys (aged 7–18 years), from schools in Tehran, Iran. Confirmatory factor analysis (CFA) was conducted to evaluate structural validity and test the fit of three-factor models. Internal consistency reliability was examined using Cronbach’s alpha and McDonald’s omega coefficients for the DASS-Y total and subscale items. Convergent and discriminant validity were also assessed.

**Results:**

The CFA results supported the original 3-factor structure of the DASS-Y, consisting of Depression, Anxiety, and Stress subscales in both groups of Iranian children and adolescents. Subsequently, the Pearson correlation coefficient assessed the scale’s convergent and discriminant validity, which was relatively appropriate. Also, the DASS-Y’s internal consistency reliability was satisfactory.

**Conclusion:**

The Persian DASS-Y is a reliable and valid Instrument for measuring depression, anxiety, and stress in Iranian children and adolescents. It can be helpful for both research and clinical work, helping to assess psychological distress in children and adolescents.

## Introduction

Childhood and adolescence are crucial stages since over 50% of mental health issues start during these years, and many persist into adulthood (Schlack et al., 2021). There is a growing concern about the increasing prevalence of anxiety, depression, and other emotional disorders in youth (Barker et al., 2019). From 2016 to 2020, around 5.8 million children and adolescents aged 3–17 years, which is 9.4% of the population, were diagnosed with anxiety. Additionally, 2.7 million children, which is 4.4% of the population, were diagnosed with depression (Lebrun-Harris et al., 2022). Anxiety, depression and stress can have serious physical, academic, and social consequences (Morales-Muñoz et al., 2023), as well as lead to self-harm, and suicide in young people (McMahon et al., 2023; Golshani et al., 2021). Therefore, it is essential to have tools that can validly and reliably evaluate anxiety and depression symptoms in children and adolescents.

The Depression Anxiety Stress Scales (DASS) and its abbreviated form, DASS-21 (Lovibond and Lovibond, 1995b; Szabó, 2010), were created to evaluate negative emotional states and used in individuals of all ages, including adults, adolescents and children. When developing the DASS scale, items related to depression were removed if they did not show sufficient consistency with the depression scale or if they could not be differentiated from the anxiety scale. Similarly, items related to anxiety were removed if they did not show adequate consistency with the anxiety scale or if they could not be differentiated from the depression scale. The construction of the DASS-Depression and DASS-Anxiety scales led to the emergence of a third factor, which was related to tension/stress, which resulted in the creation of a separate stress scale (Naumova, 2022). Thus, the Anxiety scale measures autonomic arousal, skeletal muscle effects, situational anxiety and subjective experience of anxious affect. The depression scale assesses dysphoria, hopelessness, devaluation of life, self-deprecation, anhedonia, and inertia. The stress scale measures nonspecific arousal, difficulty relaxing, irritability, and impatience (Lovibond and Lovibond, 1995a). The scales are designed to cover all aspects of fundamental symptoms associated with depression, anxiety, and stress while simultaneously providing the most significant degree of differentiation among these three states (Lovibond and Lovibond, 1995a). The abbreviated version of the DASS (DASS-21) includes only items with high factor loadings, making it an adaptation of the original DASS (Antony et al., 1998). There is sufficient published evidence supporting the use of the DASS-21 with adult populations in various clinical and non-clinical settings and across diverse cultures and nationalities. Additionally, the DASS-21 demonstrates excellent psychometric properties in adult populations, including a reliable factor structure and acceptable test–retest reliability, making it a widely preferred option among clinicians and researchers (Yeung et al., 2020). The DASS-21 is commonly used in studies involving young individuals. Studies using the DASS-21 scale on adolescents in Australia, Malaysia, Vietnam, and North Macedonia found that the scale does not discriminate well between depression, anxiety, and stress in adolescents. There are high relationships across the components in three-factor models, ranging from 0.8 to 0.9 for adolescent data; there appears to be no distinction between the constructs (Duffy et al., 2005; Hashim et al., 2011; Tully et al., 2009).

Further studies have supported a one- or two-factor structure for the DASS-21 assessment tool rather than a three-factor one. These studies have also found that it can effectively use a one-factor model to assess general distress among adolescents (Jovanović et al., 2021). Besides, evidence from cross-cultural studies suggests that the bifactor model is more effective than a three-factor model in evaluating the DASS-21 among adolescents (Shaw et al., 2017). However, in adolescents, the presentation of depression, anxiety, and stress may differ from that of adults, and the DASS scale may not help distinguish between the three states of depression, anxiety, and stress in this population (Duffy et al., 2005; Hashim et al., 2011; Patrick et al., 2010; Szabó and Lovibond, 2006). On the contrary, Some studies have supported the original three-factor model, demonstrating its fit among adolescents from various cultures, such as Australia, Chile, China, Malaysia, and Brazil (Dapieve-Patias et al., 2016; Mellor et al., 2015).

The DASS was created to measure negative emotional states in adults with clinical or general populations. However, its limited suitability has yet to be questioned regarding whether the 3-factor structure of the adult DASS can be applied to children and adolescents (Dwight et al., 2024). Particularly during the children and adolescent years, it is important to pay attention to the gradual development and differentiation of symptoms of depression, anxiety, and stress. The characteristics of depression and anxiety symptoms in young people are still not fully understood, although it is known that depression and anxiety often occur together, especially during adolescence. There are significant differences between the two conditions (Anderson and Hope, 2008; Cummings et al., 2014). Additionally, cultural differences may influence how children and adolescents express distress. This issue interested researchers in studying the cross-cultural validity of the DASS (Zahn Waxler et al., 2000). One of the unique features of DASS was the Tension/Stress subscale, which was created during the construction and development of two subscales of depression and anxiety. The researchers named a subscale “tension and stress” because it was supposed to measure something similar to the concept of stress presented by Selye (1974) general adaptation syndrome. The Tension/Stress construct of DASS-21 has acceptable validity and reliability in adult studies, its validity in studies with adolescents remains debated. Therefore, it is unclear whether children and adolescents experience tension/stress in the same way as adults, or if they respond differently to it (Szabó, 2010; Szabo and Lovibond, 2022). Furthermore, Subsequent studies in adults have shown that the DASS Tension/Stress subscale is significantly correlated with high levels of worry and Generalized Anxiety Disorder (GAD) instead of being associated with more specific fears and phobias (Brown et al., 1997; Huang et al., 2009). Although evidence exists in adult studies, our understanding of the connection between the Tension/Stress subscale and worry and GAD in children and adolescents is limited (Songco et al., 2020). Also, Questions about how worry develops in childhood and adolescence and its similarities or differences to adult worry remain controversial and require further investigation. However, Using the DASS self-report questionnaire can be challenging for young people to understand, as they may struggle to differentiate between depression, anxiety, and stress (Moore et al., 2017; Shaw et al., 2017). Therefore, it is necessary to construct alternative versions of the DASS specifically for children and adolescents in order to appropriately evaluate all three negative affective states of depression, anxiety, and stress.

In this way, Szabó and Lovibond (2006) and their research team have been working to understand the negative emotional impact on young people and develop a youth version of the DASS. Recently, Szabo and Lovibond (2022) created the Depression Anxiety Stress Scale for Youth (DASS-Y), which was administered to 2,121 Australian students aged between 8 and 17 years. The research group developed a 21-item scale from a list of 40 draft items, derived from both published and unpublished investigations. The draft list was tested on a large group of children and adolescents aged 8–17 using confirmatory factor analysis (CFA) and multiple regression analysis. After analyzing the results, it was determined that the final 21-item three-factor scale was appropriate for children and adolescents. Also, multiple regression analyses show a strong negative correlation between the DASS-Y Depression scale and positive affect and life satisfaction. The DASS-Y Anxiety scale exhibits a robust correlation with physiological hyperarousal. Another hypothesis in the study of Szabó and Lovibond was to investigate whether the DASS-Y Stress Scale is connected with high levels of worry in children and adolescents, comparable to those previously reported in adults. The findings also showed that symptoms of anxiety, depression, and stress in children and adolescents are the same as those seen in adults (Szabo and Lovibond, 2022).

A recent study was conducted on approximately 2,600 primary and middle school students from China. The study used Rasch analysis, confirmatory factor analysis (CFA), and structural equation modeling (SEM). The study’s results support the three-factor model for DASS-Y, which is consistent with the original study. The Chinese sample has shown acceptable discriminant validity, internal consistency, and convergent validity (Cao et al., 2023). Therefore, we have designed this project because there is a lack of psychometric studies of DASS-Y in the sample of Iranian children and adolescents and the necessity to develop it in the Persian language and Iranian culture. Consequently, our main objective is to explore DASS-Y’s psychometric properties and its applications among Iranian children and adolescents. Our initial hypothesis was that the three-factor structure of the DASS-Y could be proved among Iranian children and adolescents using Confirmatory Factor Analysis (CFA). Furthermore, we expected that the DASS-Y items would exhibit internal consistency and be deemed acceptable. To assess convergent and discriminant validity, we hypothesized that negative correlations would be observed among DASS-Y depression and positive affect as well as quality of life. Further, we predicted finding a robust positive correlation between DASS-Y anxiety and a measure of physiological hyperarousal. We also hypothesized that the three DASS-Y factors would display significant negative relationships with Positive affect and significant positive relationships with negative affect. Likewise, regarding the primary research on DASS-Y among Australian samples (Szabo and Lovibond, 2022), we assumed a connection between DASS-Y Stress and a measure of worry in Iranian children and adolescents. Providing evidence for these hypotheses will further reinforce the use of the DASS-Y as a reliable and valid self-report instrument to evaluate negative emotional states in Iranian children and adolescents.

## Methods

### Participants

A cross-sectional study was done during a two-month period, specifically from November to December 2023. The study protocol was authorized by the Ethics Committee of the Iran University of Medical Science (IR.IUMS.REC.1402.448). The research was carried out in accordance with the Declaration of Helsinki guidelines. Iranian children and adolescents aged 7 to 18 participated in this study. This age range was selected to encompass a critical developmental period during which individuals are most vulnerable to emotional and psychological difficulties. It allowed us to examine mental health outcomes using the DASS-Y across two key educational stages: primary school (7–12 years) and secondary school (13–18 years). These stages are characterized by significant developmental transitions, making them particularly relevant for studying depression, anxiety, and stress (Szabo and Lovibond, 2022). The study employed a combination of convenience and purposive sampling methods to recruit participants effectively. Convenience sampling facilitated the rapid recruitment of students from 15 accessible primary (ages 7–12) and secondary (ages 13–18) schools in Tehran and nearby towns through online platforms. Purposive sampling ensured the inclusion of participants who met specific criteria relevant to the study’s objectives. The eligibility requirements for participation included being between the ages of 7 and 18, residing in Tehran City, being able to understand written Persian, and having parents who consented to their child’s participation. This dual sampling approach allowed us to balance accessibility with relevance, resulting in a robust sample size suitable for analysis. Students received invitations from 15 different primary schools and secondary schools in Tehran and nearby towns. Both private, independent institutions and public, government schools provided students for recruitment. Their class instructors gave all participants the necessary information about the measurement tools and research objectives. Afterward, the teachers shared the survey link with the parents of the students through their mobile devices. Before starting the survey, the website’s homepage displayed a consent form that needed to be accepted by selecting the “Yes, I Agree” checkbox. Parents supervised the process of answering the questionnaires by their children. Also, all participants have anonymously completed the survey. A commonly cited guideline suggests a ratio of 10 respondents to 1 item. Applying this guideline to the 21-item DASS-Y would suggest a sample size of approximately 210 respondents (Boateng et al., 2018; Nunnally and Bernstein, 1978). During the survey activation period, we received 1,277 valid responses, with 562 from primary school students and 715 from secondary school students. All questions were required to complete the online survey, ensuring no missing data. Therefore, the sample used in this study is suitable for factor analysis and creating a comprehensive report.

#### Procedure

Forward and backward translation methods were used to implement the DASS-Y questionnaire in Iranian children and adolescents and adjust it to the culture and context of the Persian language. Firstly, two bilingual translators proficient in Persian independently translated the original English version resulting in versions V2 and V3. Subsequently, discussions between the corresponding author and the translators resolved any disparities, leading to a unified Persian version (V4). This version underwent further refinement by a Persian language expert (V5). Afterward, a third translator fluent in both languages translated the text back to English to produce another version (V6), facilitating comparison with the original. An expert panel comprising translators and professionals in relevant fields meticulously reviewed all versions (V1-V6) to ensure fidelity to the original and cultural relevance, culminating in the approval of the seventh Persian version (V7). The final Persian version of the DASS-Y was tested in a pilot study involving 10 primary and secondary school students. Feedback was systematically collected through interviews, focusing on the clarity, comprehensibility, and relevance of the translated items for the target age group. Based on this feedback, some items were reworded and clarified to improve comprehension and ensure that the language was accessible to children and adolescents. These adjustments were made to eliminate any ambiguities and better align the items with the constructs of depression, anxiety, and stress. After undergoing this thorough revision process, the final Persian version (V7) of the DASS-Y was developed and approved, ensuring its validity and applicability within the Persian context.

### Measures

#### Demographics

The students were requested to provide their demographic information, which includes their age and gender.

#### Depression anxiety stress scale for youth (DASS-Y)

The DASS-Y was developed by Szabó and Lovibond in 2022 as a self-report tool for assessing three subscales among youths including depression, anxiety, and stress. A DASS-Y questionnaire comprising 21 items was designed to measure the frequency of symptoms reported by participants in the past week. Participants will rate their symptoms on a 4-point scale, where 0 means not true and three means very true. In each of the three subscales, there are items like “I could not stop feeling sad” (depression), “I felt scared for no good reason” (anxiety), and “I found myself overreacting to situations” (stress). Three subscales range from 0 to 21, with a total score of 0 to 63 (Szabo and Lovibond, 2022). The scores that are higher indicate the presence of elevated levels of symptoms associated with depression, anxiety, and stress.

#### The positive and negative affect schedule for children—short form (PANAS-10)

The PANAS-10 is a short and simple self-report measure developed for evaluating children’s positive and negative affect. The positive affect items assess feelings such as excitement and joy, while the negative affect items assess feelings such as sadness and anger during the past few weeks. It consists of 10 items that children are asked to rate according to the Likert scale, which ranges from 1 (not at all or very slightly) to 5 (extremely). The ratings from the positive and negative affect items can be added independently to generate two scores, representing the child’s positive affect and negative affect, respectively (Ebesutani et al., 2012). The internal consistency in the current sample was highly acceptable for the Positive Affect Scale (*α* = 0.93) and the Negative Affect Scale (*α* = 0.92).

#### The Penn State worry questionnaire for children (PSWQ-C)

The PSWQ–C is a questionnaire designed for children and adolescents aged 7 to 17 years. It consists of 14 self-report items that measure worry intensity and uncontrollability. This instrument was originally developed by Chorpita et al., 1997. It is an adaption in accordance with the Penn State Worry Questionnaire (PSWQ; Meyer et al., 1990) for adults (Meyer et al., 1990). The questions are scored on a five-point Likert scale ranging from 1 = never to 5 = always. All items in this questionnaire are added up to determine the total score. The scale ranges from 14 to 70, with a higher score indicating higher worry levels (Chorpita et al., 1997). The measure used in this sample showed acceptable internal consistency (*α* = 0.94).

#### The physiological Hyperarousal scale for children (PH-C)

The PH-C is a scale comprising 18 self-rated items to evaluate physiological hyperarousal. Hyperarousal describes bodily indications of autonomic arousal. Children are requested to rate their experiences of autonomic arousal on a scale of 1–5, with 1 representing very slight or no experience and 5 representing extreme experience. The indicators of autonomic arousal may include sweating, shaking, and palpitations that occurred during the prior 2 weeks (range of possible scores = 18–90) (Laurent et al., 2004). PH-C demonstrated great internal consistency (*α* = 0.92) in the current sample.

### KIDSCREEN-10

KIDSCREEN-10 is a shortened version of the KIDSCREEN-27 questionnaire widely used in research and clinical settings to assess children’s subjective wellbeing and overall quality of life of young people between 8 and 18 years old. KIDSCREEN-10 is a questionnaire consisting of 10 questions that are answered using a Likert Scale ranging from 1 to 5, where 1 corresponds to “never,” 2 to “almost never,” 3 to “sometimes,” 4 to “almost always,” and 5 to “always.” The obtained scores are converted into a 0–100 scale using a linear conversion method. The higher the score, the better the quality of life is for individual youth. A Cronbach’s alpha of 0.82 and a test–retest coefficient of 0.73 are reported for internal consistency (Ravens-Sieberer et al., 2010). The measure’s internal consistency was deemed acceptable in this sample, with a coefficient alpha value of 0.75.

#### Data analysis

The data analysis was performed with the Statistical Package for the Social Sciences (SPSS version 28) and AMOS (version 24). The study’s significance level was 5.0% (*p* ≤ 0.05). Five steps were required to conduct the analysis. In the first step, descriptive data methods included frequencies, percentages, medians, ranges, means, and standard deviations. Before the analysis, the data set underwent a comprehensive screening process to identify any missing values and ensure it followed a normal distribution. The data was not missing in any instances. The properties of the DASS-Y were thoroughly examined, including the averages and standard deviations of the items, the skewness and kurtosis, and the corrected item-total correlations. The skewness (coefficient of asymmetry) and kurtosis were analyzed to evaluate the multivariate normality of the variables. Severe violations of univariate normality were detected when the skewness exceeded three and the kurtosis exceeded seven (Maroco, 2021).

Second, to examine reliability, the internal consistency of DASS-Y was assessed using SPSS.

Two methods can be used to evaluate a measure’s internal consistency: Cronbach’s Alpha and McDonald’s Omega. A Cronbach’s Alpha value above 0.70 indicates acceptable internal consistency (Nunnally and Bernstein, 1978). McDonald’s Omega is used for internal reliability, where scores of ≥0.5 are considered poor, ≥0.7 are acceptable, ≥0.8 are good, and.

≥0.9 are excellent (Hayes and Coutts, 2020).

Also, each item’s Pearson correlation was examined with the total scale to test the scale’s reliability. Correlations larger than 0.20 were preferred, and others were adjusted.

Thirdly, Confirmatory Factor Analysis (CFA) was used to evaluate the construct validity. A maximum likelihood method was used to estimate the parameter estimates. The Chi-square test is one of the most widely used methods for evaluating the fit of models to data. However, considering that the *χ*^2^ value is affected by sample size, various goodness-of-fit criteria were applied. These include the goodness of fit index (GFI), which should be ≥0.90; the comparative fit index (CFI), which should also be ≥0.90; and the root mean square residual (RMSEA) index, which should be <0.08 along with its 90% confidence interval. Other measures such as parsimony-adjusted measures (PNFI and PCFI), the HOELTER index, the expected cross-validation index (ECVI), and Akaike information criteria (AIC) are also taken into consideration. In the case of AIC, a lower value indicates a better fit (Pestana and Gageiro, 2014). For this study, only one model was tested: a three-factor structure of the 21-item DASS-Y based on the original model proposed by Szabo and Lovibond (2022). Furthermore, composite construct reliability (CCR) and average variance extracted (AVE) were calculated using the CFA approach to evaluate convergent and discriminant validity. Convergent validity was considered satisfactory if CCR was >0.70 and AVE was >0.50 for each construct and discriminant validity was confirmed when the square root of the AVE for each latent variable was greater than all correlation coefficients among those variables (Fornell and Larcker, 1981). Afterward, a CFA was conducted to examine the measurement invariance (MI) using data from the entire sample. MI was assessed across gender and age, with the final decision based on the following criteria: a change in Comparative Fit Index (CFI) of no more than 0.01 and a change in Root Mean Square Error of Approximation (RMSEA) of no more than 0.015 (Chen, 2007). Fourth, the validity of the DASS-Y construct was evaluated through a convergent validity analysis. Pearson correlations were computed between the DASS-Y and the measures indicated before to assess the scale’s convergent validity. Finally, the study utilized a known-groups validity approach to examine the relationship between DASS-Y scores and age, as well as gender differences. Specifically, an independent-sample *t*-test was conducted to compare DASS-Y subscale scores between male and female participants, assessing the scale’s ability to distinguish between groups with expected differences in depression, anxiety, and stress levels. Significant differences (*p* < 0.05) supported the validity of the DASS-Y in identifying these variations.

## Results

### Sample description

The study involved 1,277 participants, comprising 703 (55.1%) being girls and 574 (44.9%) being boys. The average age of the participants was 13.22 years, with ages ranging from 7 to 18 years old and a standard deviation of 3.07. The DASS-Y score had an average of 18.10 (SD = 10.78). Table 1 provides more detailed statistics on each item. The distribution of each item was found to be positively skewed, ranging from 0.324 to 0.773, and had a mixed kurtosis with both positive and negative values, ranging from −0.66 to 0.017. Our assumption was that any variable having a skewness ranging from −2 to +2 and kurtosis ranging from −7 to +7 was considered normally distributed (Hair et al., 2010; Shabani et al., 2018).

**Table 1:** Descriptive statistics for total items (*n* = 1,277).

DASS-21 items	Min	Max	M	SD	Mdn	Sk	Ku
1. I got upset about little thing.	0	3	1.23	0.914	1	0.445	−0.554
2. I felt dizzy, like I was about to faint	0	3	0.70	0.686	1	0.764	0.590
3. I did not enjoy anything	0	3	0.69	0.688	1	0.629	−0.212
4. I had trouble breathing (e.g., fast breathing), even though I wasn’t exercising and I was not sick.	0	3	0.71	0.694	1	0.618	−0.106
5. I hated my life	0	3	0.69	0.681	1	0.618	−0.167
6. I found myself over-reacting to situations	0	3	1.25	0.915	1	0.352	−0.661
7. My hands felt shaky	0	3	0.71	0.695	1	0.621	−0.164
8. I was stressing about lots of things	0	3	1.23	0.875	1	0.392	−0.476
9. I felt terrified	0	3	0.71	0.685	1	0.565	−0.236
10. There was nothing nice I could look forward to	0	3	0.69	0.688	1	0.565	−0.486
11. I was easily irritated	0	3	1.18	0.885	1	0.430	−0.486
12. I found it difficult to relax	0	3	1.17	0.854	1	0.381	−0.445
13. I could not stop feeling sad	0	3	0.70	0.677	1	0.637	0.058
14. I got annoyed when people interrupted me	0	3	1.15	0.839	1	0.324	−0.497
15. I felt like I was about to panic	0	3	0.72	0.699	1	0.614	−0.156
16. I hated myself	0	3	0.72	0.720	1	0.672	−0.137
17. I felt like I was no good	0	3	0.69	0.694	1	0.696	0.017
18. I was easily annoyed	0	3	1.13	0.880	1	0.481	−0.420
19. I could feel my heart beating really fast, even though I had not done any hard exercise	0	3	0.71	0.701	1	0.725	0.272
20. I felt scared for no good reason	0	3	0.65	0.678	1	0.773	0.314
21. I felt that life was terrible.	0	3	0.68	0.709	1	0.753	0.100

Min, minimum; max, maximum; M, mean; SD, standard deviation; Mdn, Median; Sk, skewness; Ku, kurtosis.

According to the cutoff values provided by the DASS-Y developers, the study aimed to determine the proportion of primary and secondary school students who showed clinically significant emotional symptoms. The results showed that a significant percentage of primary school students, 113 (20.1%), exhibited symptoms of depression, 106 (18.9%) showed symptoms of anxiety, and 33 (6%) experienced stress. In the case of secondary school students, the scores revealed a significant prevalence of depression symptoms 121 (17%), anxiety symptoms 118 (16.5%), and stress symptoms 170 (23.8%). The DASS-Y assessment tool was used to determine symptoms based on scores of 9 or higher for depression, 8 or higher for anxiety, and 14 or higher for stress (Szabo and Lovibond, 2022).

### Reliability of DASS-Y

The study used Cronbach’s alpha and McDonald’s *ω* to evaluate the internal consistency reliability of the DASS-Y questionnaire. The overall alpha value was 0.94, and the reliability scores of the three subscales ranged from 0.896 (Anxiety) to 0.918 (Depression and Stress). The McDonald’s ω indicated that the overall reliability of the DASS-Y was 0.937, while the reliability of the subscales for depression, anxiety, and stress was 0.91, 0.89, and 0.91, respectively. The corrected item-total correlation coefficients ranged from 0.636 (item 20) to 0.802 (item 11) (See Table 2).

**Table 2:** Descriptive statistics, corrected item–total correlation, factor loadings, and Cronbach’s alpha of the DASS-21 Persian version.

	Depression	Anxiety	Stress	Total
Cronbach’s alpha (a)	0.918	0.896	0.918	0.94
McDonald’s ω	0.918	0.895	0.915	0.937
Mean	4.85	4.92	8.34	18.10
SD	3.98	3.79	5.05	10.78
	Corrected item–total correlation			
Item 3	0.743			0.644
Item 5	0.760			0.659
Item 10	0.760			0.667
Item 13	0.754			0.669
Item 16	0.762			0.661
Item 17	0.743			0.652
Item 21	0.710			0.641
Item 2		0.664		0.595
Item 4		0.725		0.637
Item 7		0.728		0.633
Item 9		0.723		0.622
Item 15		0.729		0.639
Item 19		0.672		0.574
Item 20		0.636		0.570
Item 1			0.707	0.644
Item 6			0.764	0.673
Item 8			0.800	0.682
Item 11			0.802	0.690
Item 12			0.784	0.653
Item 14			0.720	0.618
Item 18			0.661	0.572

### Construct validity

The Construct validity of the DASS-Y scale was determined by using CFA, in accordance with its original structure. The model consisted of three latent variables, 21 observable variables, and 21 error terms. The results of the CFA indicated that the DASS-Y scale was a good fit for both primary and secondary school students. The model fit indices for primary and secondary school students can be found in Table 3.

**Table 3:** Model fit among three factor structures of DASS-Y.

Measure	Recommended cutoffs	21-item DASS-Y			21-item DASS-Y with correlated error		
		Primary school	Secondary school	Total group	Primary school	Secondary school	Total group
*N*		562	715	1,277	562	715	1,277
*χ*^2^(df)		663.33 (186)	1138.77 (186)	1651.11 (186)	401.65 (181)	515.44 (181)	771.26 (181)
CFI	>0.9	0.93	0.90	0.91	0.97	0.96	0.96
GFI	>0.9	0.88	0.83	0.86	0.93	0.93	0.94
NNFI	>0.9	0.91	0.88	0.90	0.94	0.95	0.95
PNFI	>0.5	0.80	0.78	0.80	0.81	0.82	0.82
PCFI	>0.5	0.82	0.80	0.81	0.83	0.83	0.83
RMSEA (90% Confidence interval)	<0.08	0.06 (0.06–0.07)	0.08 (0.08–0.09)	0.07 (0.07–0.08)	0.04 (0.04–0.05)	0.05 (0.04–0.05)	0.05 (0.04–0.05)
SRMR	<0.08	0.03	0.04	0.03	0.03	0.03	0.03
HOELTER (0.1)	>200	198	143	181	319	317	378
AIC		753.33	1258.77	1741.11	501.65	615.44	871.26
ECVI		1.34	1.76	1.36	0.89	0.86	0.68

CFI, comparative fit index; GFI, goodness of fit index; NNFI, non-normed fit index; RMSEA, root mean square error of approximation; SRMR, standardized root mean square residual; AIC, Akaike information criterion; ECVI, expected cross validation index.

While the model fit indicators existed within satisfactory thresholds, a detailed examination of modification indices was conducted for the 21-item model to detect potential misrepresentations, focusing specifically on the two distinct school groups. The modification indices identified significant error covariance between various pairs of items. Consequently, in response to these findings, the estimation of five error covariances was permitted in the final model, particularly where the indexes of modification were considerably high, and the relationship among items was logically justifiable. Consequently, the fitness indices of the 21-item DASS-Y model that had associated errors exhibited excellent adequacy across both the secondary school and primary school groups, as illustrated in Table 3.

To compare the estimated models, we assessed their fit using AIC and ECVI. The AIC and ECVI scores for the 21-item DASS-Y model were AIC = 753.33, ECVI = 1.34 for primary school students, AIC = 1258.77, and ECVI = 1.76 for secondary school students. Similarly, in the case of the 21-item DASS-Y Model that had associated error, the AIC and ECVI scores were AIC = 501.65 and ECVI = 0.89 for primary school students, and AIC = 615.44 and ECVI = 0.86 for secondary school students. Consequently, considering that lower values indicate better fit, the 21-item DASS-Y Model that had associated error emerged as the most suitable model, as depicted in Table 3.

**Figure 1:**
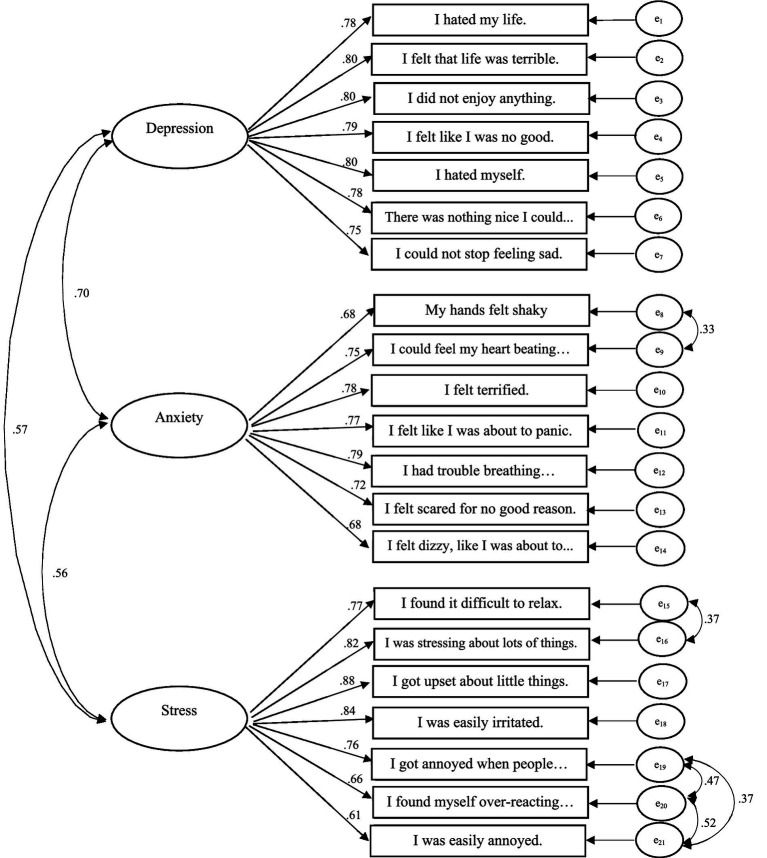
The 21-item DASS-Y model in the total sample (*N* = 1,277).

The goodness of fit values indicated a three-factor structure for the Persian version of DASS-Y. The structure was found to be adequate in the total sample, as shown in Figure 1. This attests to the factorial validity of the instrument, with GFI = 0.94, CFI = 0.96, PNFI = 0.82, PCFI = 0.83, and RMSEA = 0.05. The standardized solution of the three-factor model demonstrated that all items on the DASS-Y had a factor loading above 0.61 on their respective depression, anxiety, and stress factors. Please refer to Figure 1 for more details. The results of the bifactor model analysis for DASS-Y have been provided in the Supplementary material.

Additionally, Convergent validity was evaluated using CCR and AVE computed from factor loadings of the most suitable three-factor model. For primary school students, the CCR values stood at 0.921 for depression, 0.886 for anxiety, and 0.885 for stress, while for secondary school students, the CCR values were 0.915 for depression, 0.899 for anxiety, and 0.911 for stress. Regarding AVE values, depression (0.627), anxiety (0.529), and stress (0.528) were the AVE values for primary school students, whereas for secondary school students, depression (0.606), anxiety (0.561), and stress (0.598) were the AVE values. These findings suggest suitable convergent validity for the DASS-Y instrument.

The Fornell and Larcker (1981) criterion, assessed discriminant validity by comparing the square root of the average variance extracted ( √ AVE) for each construct on the diagonal with the correlation coefficients (off-diagonal) between constructs. This was accomplished by examining the interrelationships among the subfactors. Specifically, for primary school students, the correlation between anxiety and depression stood at 0.66, between stress and depression at 0.55, and between stress and anxiety at 0.58. Similarly, for secondary school students, the correlation between anxiety and depression was 0.61, between stress and depression was 0.57, and between stress and anxiety was 0.52. The √ AVE scores for the depression, anxiety, and stress subscales were 0.79, 0.72, and 0.72, sequentially, among primary school students, while for secondary school students, these scores were 0.77, 0.74, and 0.77, sequentially. These findings indicate that the scale’s discriminant validity was substantiated and deemed satisfactory for the measurement model.

### Invariance analysis

The initial stage involved conducting factorial invariance tests, wherein the three-factor solution was applied to the data for both male and female students (refer to Table 4). The tests included assessing the configural invariance (M1), metric invariance (M2), scalar invariance (M3), and strict invariance (M4) in a progressive manner. The results showed that the model was suitable for both genders, with M1 requiring slight adjustments and M2, M3, and M4 displaying minimal differences.

**Table 4:** Invariance analysis of the scale according to gender and age.

	*X^2^*	*df*	*X^2^/df*	CFI	RMSEA	Model contrast	△CFI	△RMSEA
Model 1								
Girls	531.84	181	2.93	0.966	0.053			
Boys	460.17	181	2.54	0.961	0.052			
M1	992.019	362	2.74	0.964	0.037			
M2	1005.57	380	2.64	0.964	0.036	M2–M1	0.000	−0.001
M3	1034.67	386	2.68	0.962	0.036	M3–M2	−0.002	0.000
M4	1112.64	412	2.70	0.959	0.037	M4–M3	−0.003	0.001
Model 2								
Younger group	380.50	181	2.10	0.969	0.050			
Older group	345.04	181	1.90	0.948	0.065			
M1	725.55	362	2.00	0.962	0.039			
M2	736.75	380	1.93	0.963	0.038	M2–M1	0.001	−0.001
M3	741.59	386	1.92	0.963	0.037	M3–M2	0.000	−0.001
M4	768.51	412	1.86	0.963	0.036	M4–M3	0.000	−0.001

M1, constrained structural weights model; M2, constrained structural intercepts model; M3, constrained structural covariances model; M4, constrained structural residuals model.

In the next stage, we tested the measurement invariance (MI) across different ages. Specifically, we divided the sample into two groups: younger adolescents aged 14–16 and older adolescents aged 17–18. The model demonstrated an excellent fit for both groups, as evidenced by the goodness of fit statistics. In particular, the configural model provided a good fit for the data, while the M3 model was also supported by a non-significant change in CFI and RMSEA values from the M1 model (ΔCFI = 0.001, ΔRMSEA = −0.002). The detailed results can be found in Table 4. Overall, the invariance tests confirmed that the model had a reasonable fit for both younger and older adolescents.

### Convergent validity

The DASS-Y was evaluated for convergent validity by calculating its Pearson correlations with other measures that assess constructs similar to those targeted by the DASS-Y in the study (according to Table 5). We also individually examined the correlation between the three DASS-Y scales in primary and secondary school students. In both primary and secondary school students, all measures were positively correlated with each other, as expected. The depression score of the DASS-Y had a higher correlation with the PANAS negative affect. In contrast, the anxiety score on the DASS-Y was slightly more strongly correlated with the Physiological hyperarousal. Lastly, the stress score of the DASS-Y was more strongly correlated with the PSWQ-C. The study found moderate to large correlations, indicating that the DASS-Y subscales’ specificity was adequate. These results showed that the DASS-Y had adequate validity and convergence.

**Table 5:** Correlations between the DASS-Y scales and other measures in primary school and secondary school students.

	M (SD)	1	2	3	4	5	6	7	8
Primary school age 7–12 (*N* = 562)									
1. DASS-Y depression	4.94 (4.05)	1							
2. DASS-Y anxiety	5.08 (3.75)	0.66	1						
3. DASS-Y stress	6.83 (4.11)	0.55	0.58	1					
4. PSWQ-C worry	17.38 (4.83)	0.60	0.56	0.73	1				
5. Physiological hyperarousal	25.52 (6.53)	0.60	0.75	0.61	0.54	1			
6. PANAS positive affect	16.10 (5.19)	−0.50	−0.39	−0.34	−0.35	−0.38	1		
7. PANAS negative affect	8.96 (3.98)	0.62	0.66	0.64	0.54	0.61	−0.32	1	
8. KIDSCREEN-10	48.88 (9.14)	−0.63	−0.50	−0.40	−0.37	−0.46	0.56	−0.42	1
Secondary school age 13–18 (*N* = 715)									
1. DASS-Y depression	4.78 (3.92)	1							
2. DASS-Y anxiety	4.80 (3.82)	0.614	1						
3. DASS-Y stress	9.52 (5.39)	0.57	0.52	1					
4. PSWQ-C worry	22.29 (7.51)	0.54	0.60	0.71	1				
5. Physiological hyperarousal	26.88 (7.40)	0.40	0.62	0.35	0.37	1			
6. PANAS positive affect	16.76 (5.12)	−0.53	−0.35	−0.43	−0.32	−0.28	1		
7. PANAS negative affect	10.14 (4.37)	0.68	0.62	0.60	0.62	0.41	−0.35	1	
8. KIDSCREEN-10	45.28 (7.10)	−0.67	−0.47	−0.52	−0.42	−0.31	0.64	−0.49	1

Correlation is significant at the 0.01 level (2-tailed). All *p* < 0.01.

PSWQ-C Worry, The Penn State Worry Questionnaire for Children. Physiological Hyperarousal, The Physiological Hyperarousal Scale for Children (PH-C). PANAS Negative Affect, The Positive and Negative Affect Schedule for Children – short form, Negative Affect scale (PANAS-10). PANAS Positive Affect, The Positive and Negative Affect Schedule for Children—short form, Positive Affect scale (PANAS-10). KIDSCREEN-10, The KIDSCREEN was designed to measure health-related quality of life (HRQoL).

### Associations between DASS-Y subscales and sociodemographic variables

The study examined the relationship between the DASS-Y and sociodemographic variables among primary and secondary school students. The results showed that age had a minimal effect on stress (*r* = 0.048), anxiety (*r* = 0.039), and depression (*r* = 0.071) among primary school students. However, among secondary school students, age had a significant impact on the DASS-Y subscale scores (*p* < 0.001) with depression (*r* = 0.163), anxiety (*r* = 0.150), and stress (*r* = 0.145) showing a positive correlation.

In order to determine criterion validity, the DASS-Y factors were compared individually among primary and secondary school students of both genders. The results indicated that there was a statistically significant difference in the DASS-Y factors between boys and girls in secondary school. However, there was no significant difference between the genders in primary school. According to Table 6, girls reported higher scores for depression, anxiety, and stress in secondary school compared to boys.

**Table 6:** Descriptive statistics of the DASS-21 subscales according to gender.

	Subscales	Boy (*n* = 254)		Girl (*n* = 308)		*t*	Cohen’s d
		M	SD	M	SD		
Primary school	DASS-Y depression	5.02	4.147	4.87	3.976	−0.426	−0.036
	DASS-Y anxiety	5.11	3.796	5.06	3.732	−0.130	−0.011
	DASS-Y stress	6.83	4.100	6.82	4.136	−0.027	−0.002
		Boy (*n* = 320)		Girl (*n* = 395)			
secondary school	DASS-Y depression	3.26	2.849	6.01	4.235	10.342^*^	0.748
	DASS-Y anxiety	3.25	3.158	6.05	3.860	10.650^*^	0.784
	DASS-Y stress	7.55	4.721	11.12	5.379	9.448^*^	0.701

M, Mean; SD, standard deviation; independent *t*-test (t); *p < 0.001.

## Discussion

The mental health of children and adolescents is a growing concern as their negative emotional state continues to worsen (Bor et al., 2014). A valid and reliable mental health assessment tool is urgently required for this population.

The DASS-21 is a tool that was initially designed for use in adults. Several studies have found that it is not suitable for children and adolescents. It is not recommended to use adult measures among children and adolescent populations (Patrick et al., 2010). However, The DASS-Y questionnaire has only been tested in certain cultures and populations, so this study aimed to translate the English version into simplified Persian and assess its psychometric properties. The Persian version was then evaluated and compared with a sample of primary and secondary school students in Iran.

In our study, we found that 20.1% of primary school students and 17% of secondary school students experienced mild to extremely severe anxiety. In comparison, 18.9% of primary school students and 16.5% of secondary school students experienced depression. Additionally, 6% of primary school students and 23.8% of secondary school students experienced stress. Previous research conducted in Iran revealed a greater prevalence of psychopathological symptoms in children and adolescents. In a recent study conducted by using DASS-21 in Iranian students, high rates of severe and extremely severe depression (16.8%), anxiety (28.3%), and stress (19.1%) were reported among adolescent females, comparable to the findings of secondary school students in our study (Hoseini-Esfidarjani et al., 2022). In keeping with these findings, a recent study performed over 5 years on a group of 5.8 million children and adolescents between the ages of 3 and 17 has reported a growing incidence of the diagnosis of anxiety and depression in this sample, at rates of 9.4 and 4.4%, respectively (Lebrun-Harris et al., 2022). These findings serve as a warning and suggest that we need to pay more attention to the mental health of children, especially adolescents. The current investigation results reveal that the DASS-Y questionnaire has a satisfactory level of internal consistency, as indicated by Cronbach’s alpha and McDonald’s omega coefficients. Other studies have found similar results demonstrating a three-factor structure, with a Cronbach’s alpha score of at least 0.80 for depression, anxiety, and stress (Cao et al., 2023; Szabo and Lovibond, 2022). These findings support the argument that the DASS-Y can be administered reliably to children and adolescents. Upon analysis of the items, it was found that all the scales had favorable discrimination indices, as indicated by the corrected item-total correlation. This shows that although the items within each subscale shared a common theme, they were not structurally or content-wise identical or interchangeable with each other. The structure of DASS-Y was evaluated through confirmatory factorial analyses, and the study’s findings suggest that DASS-Y has a three-factor structure that includes Depression, Anxiety, and Stress. These results are consistent with previous studies that used the original English version of DASS-Y (Szabo and Lovibond, 2022) and a recent study that investigated the structural aspects of the Chinese version of DASS-Y in school students (Cao et al., 2023). The results indicated sufficient convergent validity of the DASS-Y among primary and secondary school students. Depression, anxiety, and stress subscales had high composite construct reliability (CR > 0.88) and average variance extracted (AVE > 0.52). This indicates that DASS-Y can effectively measure underlying variables of mental distress, making it a suitable tool for assessing general mental health conditions. These findings align with previous research on the DASS-Y (Cao et al., 2023). To assess the discriminant validity of the DASS-Y, we conducted a thorough analysis of factor correlations and compared them to the √AVE values. Past research has highlighted unsatisfactory results (Cao et al., 2023), but our current study discovered that both primary and secondary school students exhibited satisfactory levels of discriminant validity. Nonetheless, the DASS-Y test showed some improvement in its ability to distinguish between different types of negative emotions, although it still does not meet the desired levels. This improvement has addressed the problem of the depression, anxiety, and stress subscales being highly correlated with each other. It suggests that these three subscales can now be used comparatively to evaluate different types of negative affect in individuals. The main finding of the research pertains to the invariance (in terms of configural, metric, scalar, and strict aspects) of the three-factor structure in DASS-Y scores regardless of various socio-demographic features. Based on the widely recognized CFI and RMSEA standards, these results indicate that the DASS-Y effectively measures a consistent construct regardless of age or gender. Moreover, these findings demonstrate that the scale’s units and origins remain steady, enabling assessments of inter-construct relationships and group mean comparisons. The presence of invariance between genders signifies that the structure of factors remains consistent and can be compared between boys and girls. This discovery indicates that the fundamental dimensions of negative affects, which are depression, anxiety, and stress, as represented by the DASS-Y, are equally applicable and significant to both boys and girls during their childhood and adolescent years. However, Gender invariance was found in all models (configural, metric, scalar, and strict) in the multigroup analysis, consistent with previous DASS-Y validations (Obeid et al., 2024). In our analysis of convergent validity, we found a significant correlation between the three factors represented in the DASS-Y scale. Our study also revealed a positive and significant association between Depression, Anxiety, and Stress and the negative affect of the PANAS. Conversely, we found a negative and significant relationship between these factors and the positive affect of the PANAS. These findings are consistent with prior research conducted in a diverse range of settings (Szabo and Lovibond, 2022). As expected, there is a strong correlation between the DASS-Y Anxiety subscale and Physiological Hyperarousal scores. This suggests that the DASS-Y Anxiety subscale, like the adult version, is mainly characterized by autonomic arousal symptoms and skeletal muscle effects. Also, previous studies have shown that symptoms of Physiological Hyperarousal, such as sweaty hands, feeling of choking, and heart pounding, are indicative of anxiety (Laurent et al., 2004). However, the most heavily weighted items of this construct consist of elements that reflect autonomic arousal, skeletal muscle effects, and subjective awareness of anxiety. The findings were found to be consistent with the results of the original study (Szabo and Lovibond, 2022). Furthermore, the DASS-Y Depression scale is associated with low positive affect. This finding is consistent with earlier research that considers the lack of positive affect as a distinct feature of depression among young people. It also highlights that this feature may help distinguish depressed youths and young individuals dealing with other internalizing issues such as anxiety and other negative affect (Ebesutani et al., 2012). According to the theoretical background, the present study’s findings align with Clark and Watson's (1991) tripartite model, which proposes that anxiety and depression share a common negative affect component but can be distinguished by the presence of low positive affect in depression and high physiological hyperarousal in anxiety. These findings were consistent with the tripartite model and provided further support for the convergent and discriminant validity of the DASS-Y. Moreover, depression scale scores have a strong negative correlation with quality of life scores. According to the DASS model, depression is not just a result of a lack of positive Affect. Instead, it is a complex combination that involves multiple factors that can significantly affect an individual’s quality of life, particularly among young people (Szabo and Lovibond, 2022). Depression can lead to various problems such as physical health issues, social difficulties, and poor academic performance (Morales-Muñoz et al., 2023).

The present study on Iranian children and adolescents indicates a strong correlation between worry and stress, as measured by the DASS-Y scale. This association was found to be stronger than the correlation observed between worrying and DASS-Y anxiety and depression scores. The results are consistent with previous research conducted on the DASS for adults (Brown et al., 1997; Huang et al., 2009), children, and adolescents in the original study (Szabo and Lovibond, 2022). Also, the results of the current study provide evidence that the DASS-Y stress scale is linked to excessive worrying and GAD, rather than specific fears or phobias (Szabó, 2011; Szabo and Lovibond, 2022). This implies that excessive worrying may be a more significant aspect of DASS-Y stress compared to DASS-Y anxiety and depression.

Finally, our study aimed to investigate the relationship between DASS-Y scores and sociodemographic characteristics of the sample. The findings of the study revealed that certain sociodemographic characteristics such as gender and age can be considered as risk factors for negative effects. For instance, it was observed that students who were aged between 13 and 18 had higher psychological distress scores compared to primary school students aged between 7 and 12. This finding is consistent with the results of previous studies that have explored the same topic. The results suggest that unpleasant affective states vary with age, which is consistent with the idea that there is a developmental increase in vulnerability to emotional and behavioral dysregulation in adolescence (Steinberg, 2005). Based on our research, it appears that girls tend to report higher levels of general distress, depression, and anxiety compared to boys. Furthermore, our findings suggest that older adolescents are more likely to experience general distress than younger adolescents. These results align with previous studies that have similarly concluded that adolescent girls and older age groups are more prone to depression, stress, and general distress (Tully et al., 2009).

According to the analysis of descriptive statistics, it has been observed that female students tend to have higher scores than male students across all three DASS-Y scales at the secondary school level. However, there is no significant distinction between the genders at the primary school level. This trend aligns with previous studies that suggest gender-based variances in negative emotions and associated disorders generally emerge during the adolescence years (Hale et al., 2008; Salk et al., 2017). The present study has shown an unexpected discovery that the disparities in negative emotions across genders can be partially explained by a decrease in DASS-Y scores among male students in secondary school as compared to those in primary school. It is essential to highlight that disparities between school groups must be interpreted carefully because of systematic changes. Thus, the differences in DASS-Y scores among primary and secondary school students were probably impacted by a range of factors, including age and sociodemographic characteristics, rather than being mainly determined by age.

The DASS-Y is a reliable and validated tool with consistent psychometric properties that have been tested on diverse Iranian populations, including primary and secondary school students. Our research findings support the DASS-Y’s three-factor structure, which is consistent with previous studies (Cao et al., 2023; Szabo and Lovibond, 2022). This provides further evidence that the DASS-Y is adaptable to different cultures.

### Limitations and future research

Although the Persian version of DASS-Y among children and youth underwent a comprehensive evaluation of its psychometric properties, several limitations present opportunities for future research. First, the assessments were conducted at different times among the participants, which may have led to inconsistent data collection. Second, this study did not evaluate other information about the participants’ demographic characteristics, such as their income levels, ethnic origins, or religious attitudes. Also, the presence of a diverse and multicultural population in Tehran provides a challenge to determining the effect of contextual variables on students’ resilience. Further investigations are necessary to understand how demographic factors influence grit in different societies. Third, the exclusion of participants with mental health illnesses from the study’s pool poses a challenge in determining the scale’s effectiveness in diagnosing primary and secondary school students clinically. Therefore, it is imperative that future research focuses on evaluating the psychometric traits of the DASS-Y among child and adolescent populations with clinical conditions. This will enable the adjustment of the cutoff value to improve accuracy. Fourth, Surveys were conducted with primary school students, assisted by their parents, which may have led to underreporting of symptoms. Fifth, although the sample size employed in this investigation was adequate and allowed for creating a valid and reliable scale, it is essential to emphasize that the data in this study were cross-sectional. Therefore, it is recommended that future research be conducted longitudinally to confirm the stability of the DASS-Y. The test–retest reliability of the DASS-Y was not evaluated in this study, so it cannot be confirmed that the scale is stable over time. For this reason, it is recommended that future studies be conducted to examine the temporal reliability of the DASS-Y. Sixth, it is essential to consider that selecting a convenient and non-representative sample of the Iranian population may affect a study’s conclusions and psychometric properties. Our research included an unbalanced number of male and female participants. To ensure better research outcomes in the future, it is recommended that Iranian percentile norms be sampled with a more representative sample of age and gender. Moreover, there is a need to investigate character traits and contextual variables that may have a direct influence on the occurrence of depression, anxiety, and stress symptoms among students. The final limitation is that replies may be biased due to social desirability - a common concern with self-report surveys. However, gathering data online and anonymously may mitigate this effect. Developing the scale required conducting a pilot study to confirm that the participants understood the items on the scale. However, a thorough qualitative analysis, such as in-depth interviews, is still needed. Since the target audience of the DASS-Y is children and youth and requires clear language, it would be best to conduct more rigorous qualitative research to ensure clarity. Also, it would be helpful to investigate if the same results apply to the DASS-Y, assess potential ceiling effects, and evaluate sensitivity to changes in symptom severity during treatment.

## Conclusion

With some limitations, the Persian version of DASS-Y proved reliable and valid for assessing negative emotional states in Iranian children and adolescents. Furthermore, The Persian version of the DASS-Y three-factor structure demonstrates outstanding internal consistency. Our research shows that the DASS-Y is a trustworthy brief test for measuring depression, anxiety, and stress in individuals aged 7–18. We believe that this will be helpful in both research and clinical settings.

## Data Availability

The raw data supporting the conclusions of this article will be made available by the authors, without undue reservation.
