# Supplementary material for: Exploring the psychometric properties of the Persian Depression Anxiety Stress Scale for Youth (DASS-Y): factor structure and reliability in Iranian children and adolescents
Source: Front Psychol. 2025 Jan 15;15:1452878. doi: 10.3389/fpsyg.2024.1452878 (PMC11776643; doi:10.3389/fpsyg.2024.1452878)
Supplement: Supplementary file 1 [file Table_1.DOCX]

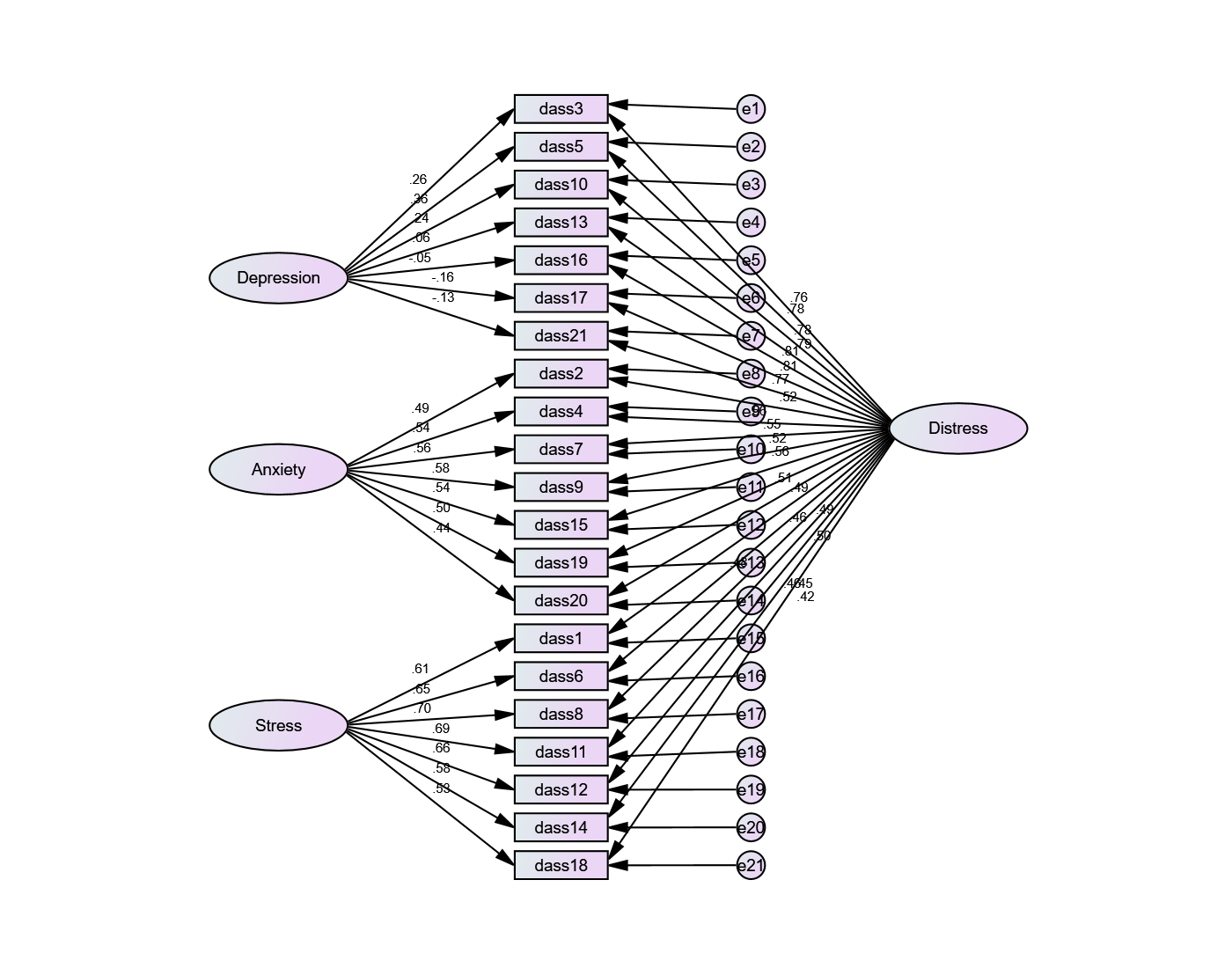


1. **Quadripartite Model of DASS-Y**


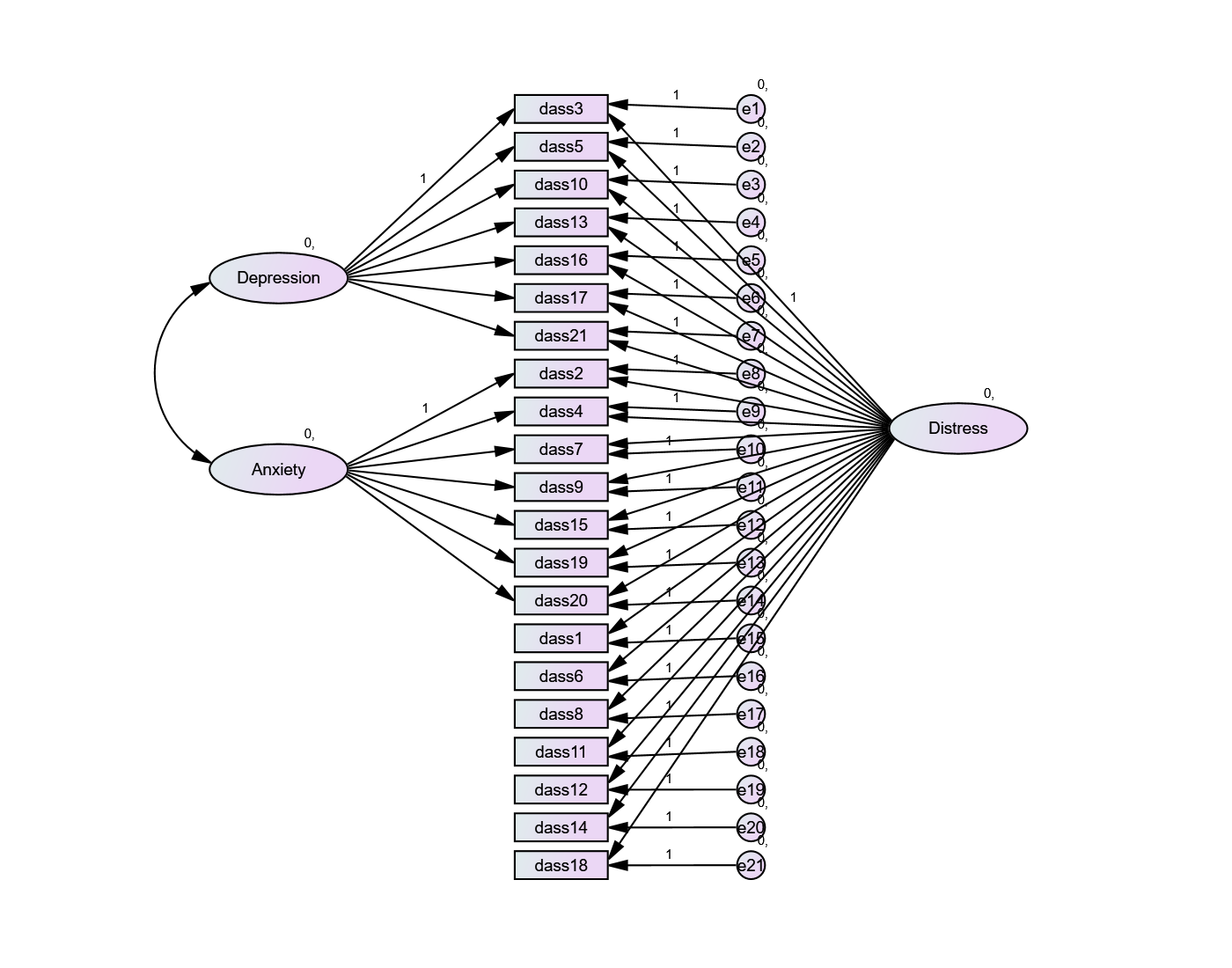


**B. Tripartite Model of DASS-Y**

Model fit among Bifactor structures of DASS-Y

| **Measure** | **Recommended Cutoffs** | **21-item DASS-Y**  **Quadripartite Model** | | |  | | **21-item DASS-Y**  **Tripartite Model** | | | | | |
| --- | --- | --- | --- | --- | --- | --- | --- | --- | --- | --- | --- | --- |
|  |  | **Primary school** | **High school** | **Total group** |  | | **Primary school** | | **High school** | | **Total group** | |
| N |  | 562 | 715 | 1277 |  | | 562 | | 715 | | 1277 | |
| χ2 (df) |  | 607.78(168) | 1095.08(168) | 1518.65(168) | | 991.06(174) | | 1520.17(174) | | 2391.77(174) | |  |
| CFI | >0.9 | .93 | .91 | .92 | | .88 | | .86 | | .87 | |  |
| GFI | >0.9 | .89 | .84 | .87 | | .85 | | .81 | | .83 | |  |
| NNFI | >0.9 | .92 | .88 | .90 | | .86 | | .84 | | .85 | |  |
| PNFI | >0.5 | .73 | .71 | .73 | | .71 | | .70 | | .71 | |  |
| PCFI | >0.5 | .75 | .72 | .74 | | .73 | | .72 | | .72 | |  |
| RMSEA  (90 Percent Confidence Interval) | <0.08 | .06 (.06-.07) | .08(.08-.09) | .07(.07-.08) | | .09(.08-.09) | | .10(.09-.10) | | .10(.09-.10) | |  |
| HOELTER (0.1) | >200 | 198 | 140 | 180 | | 125 | | 104 | | 118 | |  |
| AIC |  | 733.78 | 1221.08 | 1644.65 | | 1105.06 | | 1634.17 | | 2505.58 | |  |
| ECVI |  | 1.30 | 1.71 | 1.28 | | 1.97 | | 2.28 | | 1.96 | |  |

Notes: CFI = comparative fit index; goodness of fit index (GFI); NNFI = non-normed fit index; RMSEA = root mean square error of approximation; AIC = Akaike information criterion; and expected cross validation index (ECVI).
